# AN EPIDEMIOLOGICAL REVIEW OF OCULOFACIAL TRAUMA WITHIN A DETROIT HOSPITAL SYSTEM

**DOI:** 10.51894/001c.122838

**Published:** 2024-08-30

**Authors:** Deanna I. Miano, Parker Williams, Jeff Kuhary, Nina Singhal, Mohamad Haidar, Aishwarya Bhan

**Affiliations:** 1 Ophthalmology Ascension Macomb-Oakland

## 19

### INTRODUCTION

Incidence of orbital fractures and associated ocular injuries have proven variable amongst literature worldwide. Such an analysis has yet to be conducted in Southeast Michigan populations.

### OBJECTIVE

The primary aim of this study was to further understand the epidemiology of orbital fractures and their ocular sequelae within a Michigan population.

### METHODS

This retrospective chart review assessed incidence of ocular injuries resulting from computed tomography-confirmed orbital bone fractures from three Ascension facilities (Moross, Macomb, Oakland). Quantities were categorized based on patient demographics, injury modality, fracture type, ophthalmic morbidity, and need for surgical intervention. Ocular injury was defined as either “minor-moderate” (low threat to visual outcome), or “severe” (acutely vision threatening). Association amongst fracture and injury type was assessed. Both formal ophthalmology consultations and facial surgery consultations were required for inclusion. Leaving against-medical-advice and prior enucleation excluded subjects from review.

### RESULTS

Two-hundred and four eyes of 187 patients over 3 years and 7 months were assessed. Most fractures occurred within the inner city (55.1%), and in males (63.6%), with patient age averaging 45 years. Assault accounted for 54.6% of all injury, followed by blunt trauma (24.6%), motor vehicle accident (17.1%) and firearms (3.7%). Traumas peaked in summer months. Orbital floor fractures were most often encountered, while commonly injured bones were maxillary, ethmoid, and zygomatic bones, respectively. Most orbital fracture patients sustained some form of ocular injury (84.8%), while one-third of these patients sustained severe vision-threatening injury. Retinal detachment [p<0.0001], retinal hemorrhage [p=0.025] and sclopetaria [p=0.032] were significantly related to orbital roof fractures, while maxillary bone fractures were associated with decreased occurrence of retinal detachment [p=0.0287]. Immediate surgical repair ensued for 23% of osseous fractures, and 3.26% of ocular injuries.

### DISCUSSION & CONCLUSION

These results improve the understanding of ocular injury in the setting of concurrent orbital fractures within a Midwest population. Available data shows both differences and similarities from our own results, which may vary based on community and geographical factors. A larger analysis with incorporation of neighboring hospital systems may strengthen statistical outcomes in the future. This epidemiological data contributes to a broader understanding of Detroit’s oculofacial trauma.

